# Gender differences in HIV disease progression and treatment outcomes among HIV patients one year after starting antiretroviral treatment (ART) in Dar es Salaam, Tanzania

**DOI:** 10.1186/1471-2458-13-38

**Published:** 2013-01-15

**Authors:** Fausta Mosha, Victor Muchunguzi, Mecey Matee, Raphael Z Sangeda, Jurgen Vercauteren, Peter Nsubuga, Eligius Lyamuya, Anne-Mieke Vandamme

**Affiliations:** 1 Ministry of Health and Social Welfare, Box 65545, Dar es Salaam, Tanzania; 2 Department of Microbiology and Immunology, Rega Institute for Medical Research, KU Leuven, Leuven, Belgium; 3 Muhimbili University of Health and Allied Sciences, Dar es Salaam, Tanzania; 4 Global Public Health Solutions, Atlanta, USA; 5 Centro de Malária e outras Doenças Tropicais and Unidade de Microbiologia, Instituto de Higiene e Medicina Tropical, Universidade Nova de Lisboa, Lisbon, Portugal

## Abstract

**Background:**

We investigated gender differences in treatment outcome during first line antiretroviral treatment (ART) in a hospital setting in Tanzania, assessing clinical, social demographic, virological and immunological factors.

**Methods:**

We conducted a cohort study involving HIV infected patients scheduled to start ART and followed up to 1 year on ART. Structured questionnaires and patients file review were used to collect information and blood was collected for CD4 and viral load testing. Gender differences were assessed using Kruskal-Wallis test and chi-square test for continuous and categorical data respectively. Survival distributions for male and female patients were estimated using the Kaplan-Meier method and compared using Cox proportional hazards models.

**Results:**

Of 234 patients recruited in this study, 70% were females. At baseline, women had significantly lower education level; lower monthly income, lower knowledge on ARV, less advanced HIV disease (33% women; 47% men started ART at WHO stage IV, p = 0.04), higher CD4 cell count (median 149 for women, 102 for men, p = 0.02) and higher BMI (p = 0.002). After 1 year of standard ART, a higher proportion of females survived although this was not significant, a significantly higher proportion of females had undetectable plasma viral load (69% women, 45% men, p = 0.003), however females ended at a comparable CD4 cell count (median CD4, 312 women; 321 men) signifying a worse CD4 cell increase (p = 0.05), even though they still had a higher BMI (p = 0.02). The unadjusted relative hazard for death for men compared to women was 1.94. After correcting for confounding factors, the Cox proportional hazards showed no significant difference in the survival rate (relative hazard 1.02).

**Conclusion:**

We observed women were starting treatment at a less advanced disease stage, but they had a lower socioeconomical status. After one year, both men and women had similar clinical and immunological conditions. It is not clear why women lose their immunological advantage over men despite a better virological treatment response. We recommend continuous follow up of this and more cohorts of patients to better understand the underlying causes for these differences and whether this will translate also in longer term differences.

## Background

The widespread use of combination Antiretroviral Therapy (ART) has improved the lives of people living with HIV through reducing morbidity and mortality [1]. An infectious disease with an almost universally fatal outcome has been transformed into a manageable chronic infectious disease. Due to this, HIV testing services have expanded rapidly in many developing countries including Tanzania in order to reach ambitious targets for ART coverage [2]. However, in a substantial proportion of patients the effectiveness of ART is not sufficient with as consequence virological, clinical and immunological decay [3].

Currently, in resource limited settings, physicians start antiretroviral therapy based on the appearance of symptoms, CD4 + T-cell count and the progression of loss of CD4+ T-cells [4]. However, the success of ART in HIV infection may be influenced by numerous other factors. There is limited data presenting a combined assessment of the parameters that may affect treatment outcome in routine clinical management of HIV-infected patients in resource limited settings. It has been reported that a delay of starting ART to WHO clinical stage IV [5] or BMI below 16 kg/m^2^ is associated with a significantly higher mortality after starting treatment [6]. Within a setting of comparable clinical care, survival from the point of diagnosis of AIDS is associated mostly with the WHO stage at AIDS diagnosis, but differences in age, gender, race, and risk behaviour also exert an influence on survival [7].

The progression rates to AIDS and clinical manifestations of diseases associated with HIV infection might differ between women and men because of biological and socioeconomic factors [8]. Previous investigations found different rates of HIV disease progression and of virological and immunological response to antiretroviral therapy among HIV-infected women compared with men [9,10]. Some evidence suggests that HIV positive men have worse treatment outcomes than their women counterparts in Africa [11]. The observed differences may also be due to differences in access to ARV. In several countries, access to care and treatment is an important issue for HIV infected women, most of whom belong to ethnic or racial minorities. Males appear not to access HIV services as often as their female counterparts and also have worse treatment outcomes, including mortality. The proportion of males enrolled in ART programs in Africa is lower than females [11]. Other studies found that, women may also be less likely to start ART because they have less time to keep HIV outpatient appointments because of family commitments, fears about pregnancy, or socioeconomic circumstances [12].

In resource-limited settings, men are more likely to have more advanced disease at HIV diagnosis, which is thought to put them at higher risk of adverse outcomes and less likely to respond well to ART. Some studies found that women had higher CD4 cell count at ART initiation than men [9]. In several large HIV outcome studies from both developed countries and resource-limited settings, base-line clinical and immunologic status has been found to be a significant predictor of HIV-related morbidity and mortality. Men have also been found to have a significantly higher rate of loss to follow-up and non adherence to ART, with one study showing a direct correlation with HIV outcomes in Tanzania [13]. The clinical course in HIV infected women and men also differ because of hormones and age at the time of HIV acquisition [14]. Biologic differences between men and women have been suggested as shaping immunologic responses to ART and mortality risk.

Tanzania is among the countries where ART is currently been scaled up [2]. The main concern is whether the costly treatment programs translate to effective reduction in morbidity and mortality among patients starting ART. To date, however, to our knowledge, no studies have addressed potential gender differences in access to combination therapy and subsequent short-term prognosis in Tanzania. With growing demand for ARV drugs in Tanzania, the effect of other factors on disease progression is an important question.

Based on this we conducted a study to examine the gender difference in the disease progression and outcomes among HIV patients on ART attending a Care and Treatment Centre (CTC) in Dar es Salaam Tanzania from 2010 to 2011.

## Methods

### Setting

The study was conducted at the CTC in Temeke Municipal Hospital of Dar es Salaam region. Temeke is one of the three administrative districts of Dar es Salaam region, with an area of 656 square kilometres and is the largest district with a population of 927,310 (growth rate of 4.3% per year) [15]. Since 2005, the Centre has provided comprehensive care for almost 10,398 HIV-infected individuals. On average, 90 patients were initiated ART every month, when the CD4 counts were below 200 cells/μl or between 200–350 cells/μl when categorized with HIV defining WHO stage III or IV diseases [4].

### Study design

We conducted a cohort study in which we followed up HIV-infected ARV naïve patients for one year. All patients who were HIV positive, above 18 years of age, ARV naïve but were due to start ART were enrolled in this study upon receipt of their informed consent. We excluded patients whose medical records for the previous year could not be obtained in the clinic. Patient enrolment was done for 4 months (September to December 2010). The sample size was calculated using OpenEpi with the following assumptions, 95% significance level, 80% power, female to male ratio of 2.7:1 [16] 7.6% of men progressing to AIDS after 1 year and relative risk of 2.7 [16]. The obtained minimum sample size was 229; 67 males and 162 females.

### Data collection

A structured questionnaire translated in Swahili was used to collect all the detailed information on social demographic variables and anthropometrics information. Data with respect to HIV diagnosis, clinical and ARV treatment was collected from the patient record files and the CTC database. Patients were categorized according to the clinical and performance scale of the staging system for patients infected with HIV-1 proposed by WHO [5]. The differences on these variables were assessed between males and females. All patients were followed for a period of one year after starting standard treatment as per Tanzania guidelines [4] where treatment outcome was evaluated after 12 months from the time of initiation of therapy.

### Ethical clearance

Ethical clearance to conduct the study was obtained from the National Institute for Medical Research in Tanzania and permission to conduct the study was granted by the Hospital authority. Written Informed consent was obtained from all study participants by signing the provided consent form. Patient identifying information was de-linked from the collected data.

### Laboratory testing

Blood was collected from all the participants in EDTA collection tubes, CD4 level was estimated using Becton Dickinson FACSCalibur, and viral load using TaqMan Viral-Load Assay COBAS® AmpliPrep at the National Health Laboratory in Tanzania.

### Data analysis

Data were analyzed using *Epi Info version 3*.*5*.*1* and STATA 11. Gender differences were assessed using the Kruskal-Wallis test for continuous variables and the chi-square test for categorical data. Descriptive analysis was done for the basic demographic, clinical and immunological characteristics of patients as well as continuous variables like CD4 counts, age, plasma viral load levels and BMI for both males and females. The criterion for significance for all analyses was a two-sided p-value of less than 0.05. Survival distributions for male and female patients were estimated using the Kaplan-Meier method. Patients who were lost to follow up were censored at the date when they were last seen. Patients who were still alive at the date when the study ended were censored at this date. Survival times were expressed in days. Cox’s proportional hazards regression models were used to assess the associations between patient characteristics and outcomes. All other variables were included in multivariable models to assess their impact on the association between gender and outcomes.

## Results

### Social demographic characteristics

A total of 234 patients were recruited in this study, 164 (70%) females and 70 (30%) males with a refusal rate of 6% and 25% respectively. Not all patients eligible to start ART were enrolled in this study because not all met the inclusion criteria. Significantly more males attended secondary schools than females; had a higher income and a better knowledge on ARV use (Table 1). There was no significant difference in median age (36 years), alcohol intake, use of traditional medicine and history of use of intravenous drug of abuse (Table 1).

**Table 1:** Baseline characteristics of 234 HIV-1 infected naïve patients, Dar es Salaam, 2010

**Characteristics**	**Females (N = 164)**	**Males (N = 70)**	**P Value**
**Categorical Variables**	**N (%)**	**N (%)**	
Primary level education	156 (95.1)	59 (84.3)	**0.005**
Above 100 US Dollar monthly income	57 (34.8)	43 (61.4)	**0.0002**
Having relative to remind to take medication	133 (81.1)	59 (84.3)	0.95
Alcohol Intake	33 (20.1)	18 (25.7)	0.4
Use of traditional medicine	97 (59.2)	45 (65.2)	0.5
History of Intravenous Drug abuse	8 (4.9)	7 (10)	0.2
Knowledge on ARV use and side effects	10 (6.9)	14 (20)	**0.005**
HIV testing due to Chronic illness	105 (64)	55 (78.6)	**0.04**
Starting treatment within one year of HIV diagnosis	130 (79.3)	55 (78.6)	0.96
Presence of 2 or more opportunistic infections	75 (45.7)	37 (52.9)	0.3
WHO Staging at initiation of therapy			
Stage I	16 (9.8)	5 (7.1)	0.6
Stage II	49 (29.9)	16 (22.9)	0.3
Stage III	82 (50.0)	34 (48.6)	0.8
Stage IV	17 (10.4)	15 (21.4)	**0.04**
CD4 < 100 cells/μl at ART initiation	50 (33.3)*	31 (47.0)*	**0.05**
**Continuous variables**	**Median (IQR)**	**Median (IQR)**	**P Value**
Age (Years)	35 (30.5-43.5)	37 (33.5-42.0)	0.38
CD4 (cells/μl) at ART initiation	149 (6-148; 75-218)	102 (3-221; 47-184)	**0.02**
**Continuous Variables**	**Mean (SD)**	**Mean (SD)**	**P Value**
BMI at initiation of therapy	22 (5)	20 (4)	**0.002**
Log10 Viral Load (RNA copies/ml) at initiation of therapy	5.1 (1.3)	5.5 (1.1)	**0.05**

Legend:

*Data missing for females [14 (8.5%)] and males [4 (5.7%)].

### Clinical characteristics and history at start of ART

Significantly more males tested for HIV following a chronic illness, in contrast with females who tested without signs of AIDS. Consequently, the disease stage at HIV diagnosis was significantly more advanced in men, more men had CD4 count < 100 cells/ml at baseline, they had a significantly lower Body Mass Index and higher mean Log10 viral load (males 5.5; females 5.1) (Table 1). Most patients (79%) had been recently diagnosed (< 1 year); however the time period between HIV diagnoses to initiation of therapy was not significantly different (Table 1).

### Treatment response and disease progression

**Figure 1:**
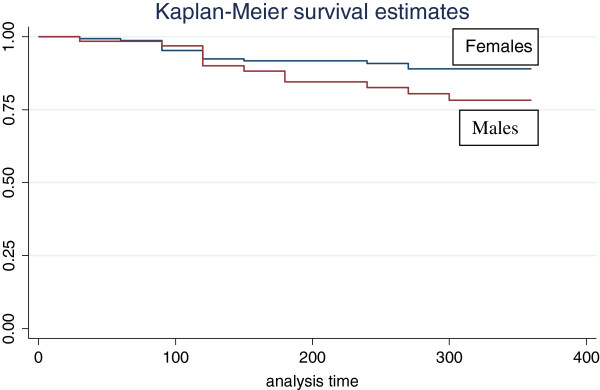
Kaplan-Meier Survival Curves on Time to Death, for 234 patients, Dar es Salaam.

**Figure 2:**
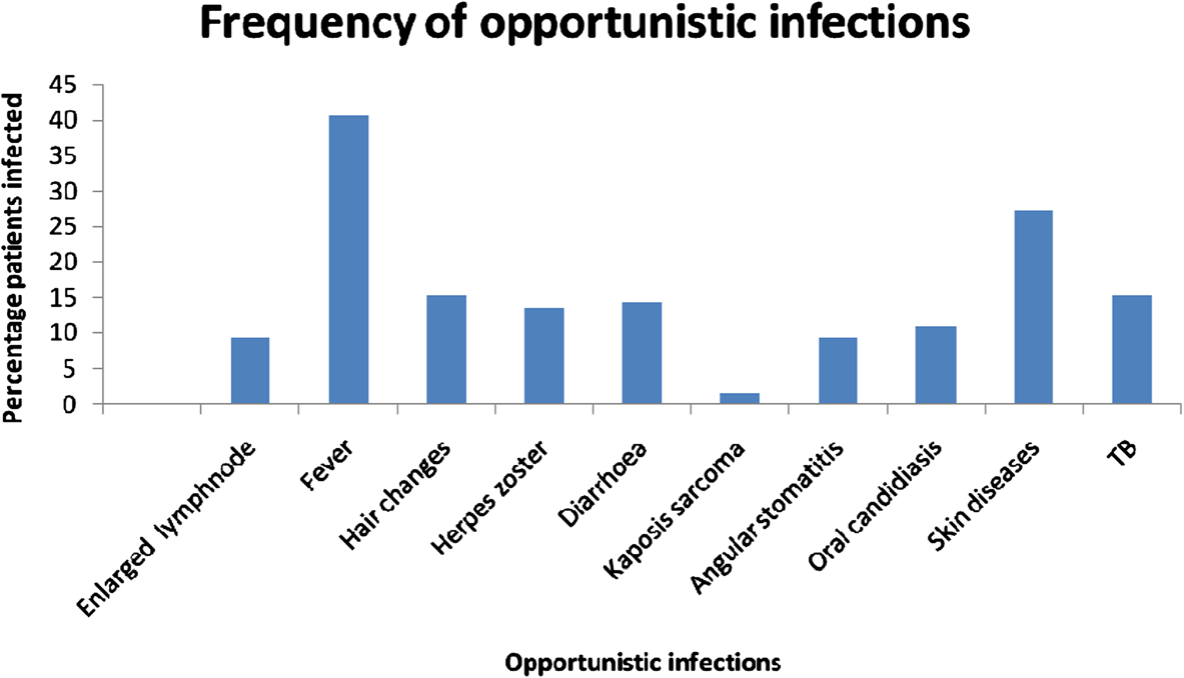
Observed Opportunistic infection during one year of follow up, Dar es Salaam Tanzania.

All the patients were receiving triple therapy combination of any of the five drugs Zidovudine, Lamivudine, Stavudine, Efavirenz and Nevirapine. There was no significant difference on the treatment regimen between men and women. The prevalence of adherence to ART as measured by consistence in keeping appointment was not different between females (62.8%) and males (62.9%) (Table 2). After one year of treatment with ART, the virological response was significantly better in females than in males (females 69%; males 45% with undetectable viral load) but the mean CD4 increase was significantly higher in males (230 cells/ml) than females (202 cells/ml) (Table 3). The BMI was still significantly higher in females (24.5) compared to males (22.5), but the percentage increase was not significantly different. Also, more females (61.6%) survived than males (50%) with more deaths occurring in males; however the difference was again not significant. The unadjusted relative hazard for death for males at 1 year of ART was 1.94 with a confidence interval of 0.91 to 4.11, p = 0.08 (Figure 1). Cox proportional hazards (of a model containing social demographic and clinical variables) showed no significant difference in the survival rate after 1 year on treatment between male and females (relative hazard 1.02, 95% CI 0.75, 1.38). The reported opportunistic infections during one year of follow up were not significantly different: mainly fever (40%), skin and hair conditions (42%), tuberculosis (15%), diarrhoea (15%) and Herpes Zoster (14%), (Figure 2).

**Table 2:** One year Outcomes of 234 HIV infected patients after starting ART from September 2010 to August 2011

**Characteristics**	**Females (N = 164)**	**Males (N = 70)**	**P Value**
	**N (%)**	**N (%)**	
Death	21 (12.8)	14 (20)	0.2
Alive	101 (61.6)	35 (50.0)	0.1
Lost to follow up	42 (25.6)	21 (30.0)	0.5
Missed appointments	103 (62.8)	44 (62.9)	0.99

**Table 3:** Progression of patients one year on ART, Dar es Salaam

**Characteristics**	**Females (N = 101)**	**Males (N = 53)**	**P Value**
Percentage with undetectable viral load	70 (69%)	24 (45%)	**0.003**
**Continuous variables**	**Median (IQR)**	**Median (IQR)**	
CD4 (cells/μl) count after 1 year	312 (252-413)	321 (110-480)	0.6
**Continuous Variables**	**Mean (SD)**	**Mean (SD)**	
Percentage BMI increase (from Baseline)	10.5 (14.2)	9.8 (17.5)	0.3
BMI after 1 year	24.5 (4.8)	22.5 (4.1)	**0.02**
Percentage weight gain	10.4 (14.3)	9.3 (17.3)	0.2
CD4 (cells/μl) increase from baseline	202 (516; 35-163)	230 (272; 86-181)	**0.05**

## Discussion

Our analysis of gender difference found significant clinical and social-demographic variations between females and males. Similar as in other studies, males were reporting for care with a more advanced disease than females. Sex differences in health seeking behaviour are known to exist as indicated in several studies [17]. This can be attributed, in part; due to the fact that females are having extra entry points to HIV services e.g. through PMTCT services, however this was not the case in this study, where the majority of the patients were tested after a long term illness. The most common reason for HIV testing was AIDS related syndrome, more than voluntary testing, and this was more pronounced in males, denying the patients time for care at CTC prior to ART.

While there was a difference in disease stage at registration for care at CTC, both genders were presenting late and there was not much difference on the period of illness before starting ART between the two groups. Males however were better informed about the use of ARVs than females. For both males and females, late presenting resulted in a median CD4 at initiation of therapy below 150 cells/ml, where in ideal situation the majority of patients on follow up will start ART with a higher CD4 count and a less advanced disease. Starting ART with low CD4 counts has been shown to be associated with early mortality mainly caused by immune Reconstitution Inflammatory Syndrome (IRIS), which appears after starting ART at advanced HIV disease (WHO stage IV), CD4 count below 50 cells/ul and BMI below 16 kg/m^2^[4] which could also have happened to some of the patients in this cohort. Another study has indicated that a low CD4 cell count at ART initiation was a strong predictor of mortality [18] and this could be the case in our study.

Despite the facts that all the patients were prepared to start ART, there were still a significant proportion of patients who were using traditional medicine, alcohol and injection drug abuse. The history of using traditional medicine and alcohol was high among our study participants, particularly among males. This may also be a reason of delaying medical treatment until late at WHO stage III and stage IV when the patients had already developed opportunistic infections. Injection drug use (IDU) is also associated with both, non-adherence to ART and HIV disease progression, and many IDU live in unstable housing, have undiagnosed mental illness, high rates of incarceration, and street-involved survival-lifestyles, which may all complicate delivery of HIV-related treatments [19].

Overall, females were found to start ART at a less advanced disease stage, with higher CD4 count and higher BMI, and with lower viral loads than males. Similar findings are reported by other studies [9]. Given the high proportion of drug abuse and alcohol in our study, our patients may have been predisposed to poor adherence [20]. There was no significant difference on ART adherence as measured by consistency on keeping appointments, but it was indeed low in both groups. Drug and alcohol consumption may also influence survival of HIV-infected individuals by exacerbating immunosuppression, enhancing the toxicity of ARV on liver cells and accelerating liver damage and may depress the immune system leading to increased multiplication of the virus in mononuclear cells [21]. After one year of treatment, immune recovery was good in both males and females; however, despite the better start for women, there was no difference in clinical (including survival) or immunological condition at one year. Although a higher proportion of males died (not statistically significant), the males who survived were found to have significantly higher mean CD4 increase than females, despite a better virological treatment response in women. This is in contrast with the findings of other studies which found better survival, less disease progression and better immunological recovery among females on HAART [10,16,22]. The difference could be due to a shorter follow up (one year) in our study, a bias among the high proportion of patients lost to follow-up or true differences in our setting. The fact that men recovered quickly during ART, despite their late presentation is encouraging. It is however unclear why the women in our study lost their advantage so quickly, which is in contrast with other studies. It may be related to the fact that they are less educated with lower monthly income, as it has been found through other studies in Tanzania [13].

The differences that we found will need further evaluation as this may need redefining the time to initiate ART in the two groups and the methods to monitor treatment response. The possibility of initiating ART at lower viral loads in women, especially during the early stages of infection, merits further study. Although the relative viral load has a similar predictive value for progression to AIDS for men and women, the same absolute viral load seems to confer different risks for AIDS between the sexes [17]. Because manifestations of HIV infection stem from the interplay between viral and host factors, sex differences in immune modulation will likely play instrumental roles in determining the course of disease. Both groups reported opportunistic infections; but more fever and oral candidiasis was reported by females than males, however, the difference was not statistically significant. Because of the late presentation to CTC, care and treatment services like prophylaxis against opportunistic infections may be suboptimal.

Our study findings are limited by the following issues: our cohort was rather small and may not necessarily represent all HIV-infected patients in need of treatment. We also missed some of the clinical information that may be useful on comparing the differences between females and males HIV patients. Due to the high number of Lost to follow-up (LTF), mortality is likely underestimated.

## Conclusion

We assessed the gender differences on HIV disease progression and outcomes after one year of ART among HIV infected patients and whether this potential difference is influenced by socio-demographic, clinical, immunological and virological differences among patients starting ART in a hospital in Dar es Salaam. Male HIV patients delay seeking treatment and enter into treatment at a significantly more advanced stage of HIV infection, which predisposes them to increased mortality and worse treatment outcome. However, we observed that after one year of ART, males and females had similar clinical and immunological conditions. It is not clear why women lose their immunological advantage over men already at 1 year of treatment despite a better virological treatment response. It may be related to the study design, to the fact that women had a lower socio-economical status or to biological differences. We recommend continuous follow up of this and more cohort of patients to understand responses to ART and the differences between males and females, together with advocating early HIV diagnosis and treatment.

## Abbreviations

AIDS: Acquired immunodeficiency syndrome; ART: Antiretroviral treatment; ARV: Antiretroviral; BMI: Body mass index; CTC: Care and treatment centre; HAART: Highly active antiretroviral therapy; HIV: Human immunodeficiency virus; IDU: Injection drug users; LTF: Lost to follow up; PMTCT: Prevention of mother to child transmission; WHO: World Health Organization.

## Competing interests

The authors declare that they have no competing interest.

## Authors’ contribution

FM designed the study, performed data analysis and wrote the first draft of the manuscript, VM conducted the interviews and supervised laboratory work together with manuscript review, MIM assisted study design and supervised interviews and clinical examination and manuscript review, RZS assisted in supervising laboratory work and manuscript review, JV and PN assisted data analysis and manuscript review, EL and AV supervised the overall study implementation and manuscript development process. All authors read and approved the final manuscript.

## Pre-publication history

The pre-publication history for this paper can be accessed here:

http://www.biomedcentral.com/1471-2458/13/38/prepub
